# Complement protein C1q stimulates hyaluronic acid degradation *via* gC1qR/HABP1/p32 in malignant pleural mesothelioma

**DOI:** 10.3389/fimmu.2023.1151194

**Published:** 2023-06-02

**Authors:** Andrea Balduit, Romana Vidergar, Paola Zacchi, Alessandro Mangogna, Chiara Agostinis, Micaela Grandolfo, Cristina Bottin, Francesco Salton, Paola Confalonieri, Andrea Rocca, Fabrizio Zanconati, Marco Confalonieri, Uday Kishore, Berhane Ghebrehiwet, Roberta Bulla

**Affiliations:** ^1^ Institute for Maternal and Child Health, Istituto di Ricovero e Cura a Carattere Scientifico (IRCCS), Burlo Garofolo, Trieste, Italy; ^2^ Department of Life Sciences, University of Trieste, Trieste, Italy; ^3^ Neuroscience Area, International School for Advanced Studies (SISSA), Trieste, Italy; ^4^ Department of Medical, Surgical and Health Science, University of Trieste, Trieste, Italy; ^5^ Struttura Complessa di Anatomia ed Istologia Patologica, Azienda Sanitaria Universitaria Giuliano Isontina (ASUGI), Trieste, Italy; ^6^ Department of Veterinary Medicine, United Arab Emirates University, Al Ain, United Arab Emirates; ^7^ Department of Medicine, Stony Brook University, Stony Brook, NY, United States

**Keywords:** hyaluronic acid, hyaluronidase, C1q, HYAL2, gC1qR/HABP1/p32, reactive oxygen species, malignant pleural mesothelioma

## Abstract

Complement component C1q can act as a pro-tumorigenic factor in the tumor microenvironment (TME). The TME in malignant pleural mesothelioma (MPM) is rich in C1q and hyaluronic acid (HA), whose interaction enhances adhesion, migration and proliferation of malignant cells. HA-bound C1q is also capable of modulating HA synthesis. Thus, we investigated whether HA-C1q interaction would affect HA degradation, analyzing the main degradation enzymes, hyaluronidase (HYAL)1 and HYAL2, and a C1q receptor candidate. We first proceeded with the characterization of HYALs in MPM cells, especially HYAL2, since bioinformatics survival analysis revealed that higher *HYAL2* mRNA levels have an unfavorable prognostic index in MPM patients. Interestingly, Real-Time quantitative PCR, flow cytometry and Western blot highlighted an upregulation of HYAL2 after seeding of primary MPM cells onto HA-bound C1q. In an attempt to unveil the receptors potentially involved in HA-C1q signaling, a striking co-localization between HYAL2 and globular C1q receptor/HABP1/p32 (gC1qR) was found by immunofluorescence, surface biotinylation and proximity ligation assays. RNA interference experiments revealed a potentially regulatory function exerted by gC1qR on HYAL2 expression, since *C1QBP* (gene for gC1qR) silencing unexpectedly caused HYAL2 downregulation. In addition, the functional blockage of gC1qR by a specific antibody hindered HA-C1q signaling and prevented HYAL2 upregulation. Thus, C1q-HA interplay is responsible for enhanced HYAL2 expression, suggesting an increased rate of HA catabolism and the release of pro-inflammatory and pro-tumorigenic HA fragments in the MPM TME. Our data support the notion of an overall tumor-promoting property of C1q. Moreover, the overlapping localization and physical interaction between HYAL2 and gC1qR suggests a potential regulatory effect of gC1qR within a putative HA-C1q macromolecular complex.

## Graphical Abstract

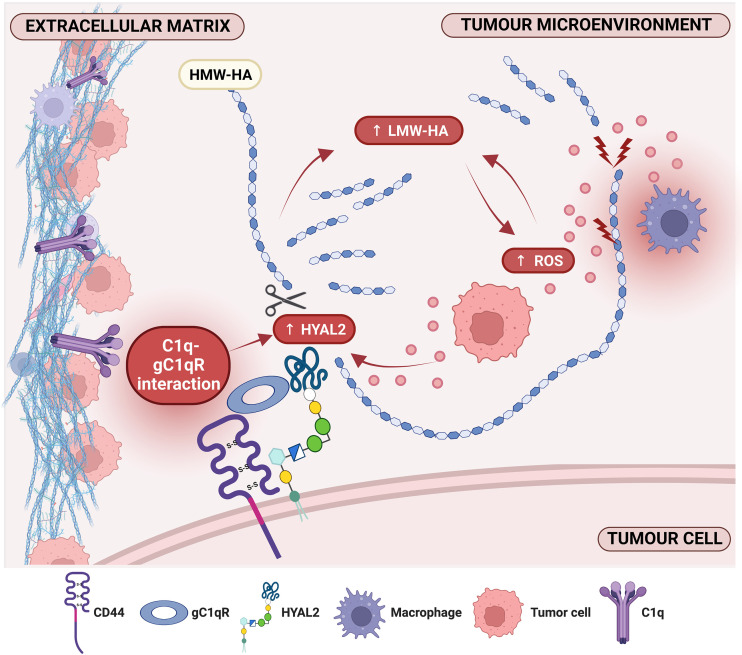

## 1 Introduction

Malignant pleural mesothelioma (MPM) is a rare and aggressive tumor of the pleural lining, primarily associated with asbestos exposure. Owing to the non-specific and late-onset symptoms, as well as the long latency period, its diagnosis is usually delayed and patient survival is undermined by the absence of efficient first-line therapeutic options (1). The development of chemoresistance is another challenge for clinicians, with almost 50% of cases showing resistance to treatments (2).

The tumor microenvironment (TME) plays a critical role in tumor progression by providing a permissive *niche* for tumor cell survival, growth and migration. As a major component of the extracellular matrix, hyaluronic acid (HA), as well as the enzymes responsible for its metabolism, are implicated in the regulation of several aspects of tumorigenesis (3, 4). In its native form, HA is a high molecular-weight (HMW) polymer synthesized by three plasma membrane hyaluronan synthases (HAS1, HAS2, HAS3), and degraded enzymatically by hyaluronidases (HYALs) (5), or non-enzymatically by oxidative stress (6).

Among the six HYALs present in humans, HYAL1 and HYAL2 are the main contributors to HA catabolism in somatic tissues; HYAL3 and HYAL4 lack intrinsic hyaluronidase activity; HYAL5 (also known as PH-20 or SPAM-1) is a sperm-specific enzyme, whereas HYAL6/HYALP1 is a pseudogene (5, 7). Recently, novel proteins have been identified for their extracellular HA-degrading capacity, namely CEMIP/KIAA1199 and TMEM2 (8, 9).

HYAL2, a glycosylphosphatidylinositol (GPI)-anchored membrane receptor, binds and cleaves HMW-HA into low molecular-weight HA (LMW-HA; ~ 20 kDa) fragments at the cell membrane (10). This mechanism has been proposed to require the presence and activity of the major HA receptor CD44, which is essential for intracellular signaling (11). Inside lysosomes, HA is further processed into oligomeric HA fragments by HYAL1, together with exoglycosidases (12). HYAL activity is fundamental since the molecular size of HA significantly impacts on its biological roles: HMW-HA exerts anti-inflammatory, anti-proliferative and anti-angiogenetic functions, while LMW-HA fragments induce signaling cascades commonly associated with inflammation, angiogenesis and diminished tumor immune surveillance (13). HA size-dependent signaling acts as a double-edged sword relying on the different proteins HA is able to interact with, namely hyaladherins or hyaluronan-binding proteins (14). Both HA and the enzymes involved in its metabolism have been frequently associated with cancer progression, leading to an overall increased HA turnover (15). In particular, deregulation of HYAL expression has been associated with different outcomes in several solid tumors (7).

The complement system (C), a powerful arm of the innate immunity, represents a key player in the TME, exerting pro- or anti-tumorigenic effects depending on the cancer type (16). In particular, the first recognition subcomponent of the classical pathway, C1q, has emerged as a cancer promoting factor independent of C activation (17). Further *in silico* studies revealed a more complex *scenario* for C1q role in cancer, unveiling its potential prognostic implications in carcinomas (18) and gliomas (19). C1q is highly expressed in several solid tumors, including MPM (20), where it strongly binds to HA, enhancing tumor cell proliferation, adhesion and migration in MPM (20).

Interestingly, the receptor for the globular head of C1q (gC1qR), also called HA-binding protein 1 (HABP1) or p32, is able to bind both C1q and HA by virtue of its pleiotropic nature (21). Despite being predominantly localized in the mitochondria (22), gC1qR may also be found in other cellular compartments (*i.e.*, endoplasmic reticulum, nucleus) and on the cell membrane (23), suggesting its important role as a modulator of different ligands both inside and outside the cell. Thus, emerging non-immune functions of gC1qR have been investigated in recent years, including its involvement in tumor cell proliferation, migration, and immune modulation (24). gC1qR overexpression has been documented in a variety of cancers, including MPM (25, 26).

Since HA-bound C1q is capable of modulating HAS expression (27), we aimed at determining whether C1q would impact also on HYAL expression, focusing on the main degradation enzymes, HYAL1 and HYAL2. Moreover, we investigated the involvement of gC1qR as a potential receptor involved in the process.

## 2 Material and methods

### 2.1 Reagents and antibodies

HA was kindly provided by Prof. Ivan Donati (Department of Life Sciences, University of Trieste, Italy). The following antibodies were used: mouse monoclonal anti-lysosomal-associated membrane protein 1 (LAMP1) and mouse monoclonal anti-actin were purchased from Santa Cruz Biotechnology (Santa Cruz, CA, USA); mouse monoclonal anti-early endosome antigen 1 (EEA1), mouse monoclonal anti-calnexin, mouse monoclonal anti-mannose 6-phosphate receptor (M6PR), anti-mouse Alexa Fluor 594-conjugated antibody, anti-rabbit Alexa Fluor 488-conjugated antibody, rabbit polyclonal anti-HYAL1 (#PA5-79420), rabbit polyclonal anti-HYAL2 (#PA5-24223) and mouse monoclonal anti-CD44 (#MA513890) from Invitrogen (Thermo Fisher Scientific, Waltham, MA, USA); rabbit polyclonal anti-HYAL2 (#15115-1-AP) from Proteintech (Proteintech, Rosemount, IL, USA); donkey anti-rabbit HRP-conjugated secondary antibody from Sigma (Sigma-Aldrich, Saint Louis, MO, USA); anti-mouse LI-COR IRDye 680RD and anti-rabbit LI-COR IRDye 800CW from LI-COR Biosciences (LI-COR Biosciences, Lincoln, NE, USA); mouse monoclonal anti-gC1qR 74.5.2, rabbit polyclonal anti-gC1qR and rabbit polyclonal F(ab**’**)_2_ anti-gC1qR were obtained as described earlier (28).

Recombinant human C1q was purchased from Sigma-Aldrich (#C1740). All other chemicals were purchased from Sigma-Merck.

### 2.2 Patients and specimens

Patients included in the study were enrolled at the Department of Pneumology, University Hospital of “Cattinara” (Trieste, Italy); they had a history of asbestos exposure and symptoms suggestive of MPM. Patients underwent video-assisted thoracoscopy/pleuroscopy for pleural biopsy sample collection, as previously described (20). After histological confirmation of MPM, a cohort of ten patients was selected: all patients were male, Caucasian, presented epithelioid histotype; none of them received chemotherapy or radiotherapy prior to biopsy sampling. The mean age at diagnosis was 74.2 ± 6.0 years. All patients signed an informed consent form, following approval of ethical considerations by the Comitato Etico Unico Regionale (CEUR, FVG, Italy; number 34/2016).

### 2.3 Cell isolation and culture

Primary MPM cells were isolated from pleural biopsies, as previously described (20), and cultured in Human Endothelial Serum Free Medium (HESFM, Gibco, ThermoFisher) supplemented with 20 ng/mL of epidermal growth factor (Società Italiana Chimici, Life Sciences), 10 ng/mL basic fibroblast growth factor (Società Italiana Chimici, Life Sciences), 1% penicillin-streptomycin (Sigma-Aldrich) and 10% heat-inactivated fetal bovine serum (FBS, Gibco, ThermoFisher). Cells were cultured at 37°C in 5% v/v CO_2_ incubator and the medium was changed every 2-3 days. To determine the purity of MPM primary cells, cytofluorimetric analysis and immunofluorescence assays were performed using the following markers: mesothelin, calretinin, cytokeratin 8/18, WT1 and CD44. In addition, CD45 and von Willebrand Factor were used to exclude leukocyte and endothelial cell contamination, respectively (20). H28 cells, a human MPM cell line, were purchased from the ATCC (#CRL-5820) and cultured in RPMI-1640 medium supplemented with 10% FBS. *ZL34*, a human MPM cell line, was kindly provided by Prof. Ioannis Kalomenidis (National and Kapodistrian University of Athens, Evangelismos Hospital, Athens, Greece) and was cultured in RPMI-1640 medium supplemented with 10% FBS.

### 2.4 Zymography

A 10% acrylamide gel impregnated with 0.17 mg/mL of HMW-HA (1.5 MDa) was prepared. Cell lysates were mixed with 2X sample buffer containing no reducing agents (20% glycerol, 63 mM Tris-HCl, pH 6.8, 2% SDS, 0.1% bromophenol blue) and loaded. Human serum was loaded as a positive control for acidic HYALs. SDS-PAGE was run at room temperature (RT) under constant current conditions (20 mA). Following electrophoresis, gel was incubated with 2.5% Triton X-100 with agitation for 1h at RT, in order to remove SDS, and then in a hyaluronidase assay buffer (100 mM sodium formate, 0.15 M NaCl, pH 4.0) for 16h at 37°C. Gel was transferred for 2h at 37°C to a buffer made up of 100 mM Tris-HCl, 20 mM NaCl, pH 8.0, containing 0.1 mg/mL pronase. Fixation of the gel was carried out using 20% ethanol and 10% acetic acid for 20 min at RT, followed by staining with 0.5% Alcian blue in 20% ethanol and 10% acetic acid for 1h at RT, and de-staining with a solution containing 20% ethanol and 10% acetic acid for 1h at RT.

### 2.5 Immunohistochemical analysis

MPM tissue samples were fixed in 10% *v/v* buffered formalin and then paraffin-embedded. Four µm-tissue sections were deparaffinized and rehydrated. The antigen unmasking technique was performed, using citrate buffer, pH 6, or Tris-EDTA buffer, pH 9, in thermostatic bath at 98°C for 30 min. Sections were then allowed to reach RT and washed in PBS. After neutralization of endogenous peroxidase activity with 3% *v/v* H_2_O_2_ and blocking of non-specific bindings by PBS + 2% bovine serum albumin (BSA), samples were incubated with HYAL1 and HYAL2 primary antibodies overnight (O/N) at 4°C. Staining was revealed *via* anti-rabbit HRP-conjugated secondary antibody and 3-amino-9-ethylcarbazole (AEC, Dako, Agilent, Denmark) substrate chromogen. Slides were then counterstained with Mayer Hematoxylin (Diapath, Italy).

### 2.6 Microtiter coating of HMW-HA and C1q

Cell culture plates were incubated O/N at 4°C with HMW- HA (MW 1.5 MDa) at the concentration of 50 µg/mL in carbonate/bicarbonate buffer, pH 9.6. The following day, the plate was washed with PBS and then incubated O/N at 4°C or for 2h at 37°C with C1q at the concentration of 25 µg/mL in 0.5% BSA in dPBS, containing 0.7 mM CaCl_2_ and 0.7 mM MgCl_2_. Wells were then washed again with PBS, before seeding the cells.

### 2.7 RT-qPCR analysis

MPM primary cells (1x10^6^) were seeded onto HA alone or HA+C1q-coated 6-well plates and collected after O/N incubation at 37°C in 5% *v/v* CO_2_ incubator. An untreated sample was collected as a control. After centrifugation, cell pellets were resuspended in RNA Lysis Buffer and mRNA was isolated from cell lysates using RNA purification kit (Norgen Biotek company, Thorold, ON, Canada). Isolated mRNA was then converted into cDNA using SensiFAST™ cDNA Synthesis Kit (Meridian Bioscience, Memphis, TN, USA).

For Real-Time quantitative PCR (RT-qPCR), SYBR Select Master Mix (Applied Biosystems, ThermoFisher) was used. The reaction was performed using Rotor-Gene 6000 (Corbett, Explera), following a program of 45 cycles of denaturation (60 sec at 95°C), annealing (30 sec at 60°C) and amplification (60 sec at 72°C). Expression levels of the genes of interest were evaluated with a comparative quantification based on the reaction efficiency and normalized against a housekeeping gene, TATA-Box Binding Protein (TBP), which is constitutively expressed in tumor cells. Primers used for RT-qPCR analysis are listed in **Table 1**.

**Table 1:** Primers used for quantitative RT-qPCR.

Gene	Melting Temperature (°C)	Forward sequence Reverse sequence	Accession Number
***HYAL1***	59	CGATATGGCCCAAGGCTTTAG ACCACATCGAAGACACTGACAT	NM_153282.2
***HYAL2***	60	GGCCCCACCGTTACATTGG ATTCTGGTTCACAAAACCCTCAT	NM_003773.5
***C1QBP***	60	ACAACAGCATCCCACCAACAT ATGACAGTCCAACACAAGGGC	NM_001212.4
***CD44***	60	CTGCCGCTTTGCAGGTGTA CATTGTGGGCAAGGTGCTATT	NM_000610.4
***TBP***	60	GAGCCAAGAGTGAAGAACAGTC GCTCCCCACCATATTCTGAATCT	NM_003194.4

C1QBP, C1q binding protein; HYAL, hyaluronidase; TBP, TATA-box binding protein.

### 2.8 Flow cytometry

MPM primary cells (5x10^5^) were fixed in 3% *v/v* paraformaldehyde (PFA) for 20 min in dark, and then incubated with anti-HYAL1 and HYAL2 primary antibodies diluted in the Permeabilization reagent of FIX & PERM kit (Invitrogen, ThermoFisher), for 1h on ice. Incubation with FITC-conjugated secondary antibodies was performed for 30 min on ice, in dark. Cells were resuspended and fixed in 1% PFA. Fluorescence was acquired using FACScalibur (BD Bioscience) and data processed using the FlowJo 10.8.2 software.

### 2.9 Immunofluorescence

MPM primary cells (5x10^4^) were seeded onto round glass coverslips and grown to 70% confluence. Cells were fixed with 3% PFA for 20 min at RT, in dark. Permeabilization, quenching and blocking were performed by incubating the cells in 1% BSA, 0.1% Triton X-100 and 50 mM glycine in PBS, for 30 min at RT. Incubation with primary antibodies, diluted in 2% BSA in dPBS, was carried out O/N at 4°C. Anti-HYAL2 (1:50), anti-EEA1 (1:200), anti-LAMP1 (1:50), anti-M6PR (1:1000), anti-calnexin (1:1000), anti-gC1qR (1:100), were used as primary antibodies.

The following day, incubation with Alexa Fluor 488 or 594-conjugated secondary antibodies (1:300) was carried out for 30 min at RT. Nuclei were stained with DAPI (Sigma-Aldrich, 1:1000) for 5 min. Glasses were mounted with Fluorescence Mounting Medium (Dako). Images were acquired by the confocal microscope Nikon A1R, SISSA facility.

### 2.10 Bioinformatics analysis

Survival analyses were performed with GEPIA2 (http://gepia2.cancer-pku.cn) using data from mesothelioma’s The Cancer Genome Atlas (TCGA-MESO) dataset, collecting mRNA from 82 MPM patients (29). Patients’ cut off was set at the median. All the results were displayed with p-values from a log-rank test. P-value (*p*) < 0.05 was considered significant.

### 2.11 Western blot analysis

MPM primary cells (1x10^6^) were seeded onto HA- or HA+C1q-coated 6-well plates and collected after O/N incubation at 37°C in 5% *v/v* CO_2_ incubator. An untreated sample was collected as a control. Cells lysates were fractioned by 10% SDS-PAGE under reducing conditions and transferred to a nitrocellulose membrane using the semi-dry transfer apparatus Trans-Blot Turbo System (BIO-RAD). After 1h of incubation with 5% skimmed milk in TBST (10 mM Tris, pH 8.0, 150 mM NaCl, 0.5% Tween 20), the membrane was probed with anti-HYAL1, anti-HYAL2, anti-gC1qR, anti-CD44 and anti-actin antibodies O/N at 4°C. Membrane was washed three times for 5 min, and then incubated with LI-COR IRDye secondary antibodies for 1h at RT. After three washing steps, the fluorescence intensity was assessed by the Odyssey^®^ CLx near-infrared scanner (LI‐COR Biosciences, Lincoln, NE, USA). Image acquisition, processing and data analysis were performed using Image Studio 5.2 (LI-COR Biosciences).

### 2.12 Surface biotinylation assay

Primary MPM cells (1x10^6^/well) were seeded onto HA- or HA+C1q-coated 6-well plates. After O/N incubation, cells were washed with ice-cold PBS supplemented with 1 mM MgCl_2_ and 0.1 mM CaCl_2_ in order to remove contaminating proteins. Cells were then incubated with EZ-LinkTM- Sulfo-NHS-Biotin (1 mg/mL) for 30 min on ice on an orbital shaker. To remove the excess of biotin, quenching was performed with 0.1 M glycine for 5 min at RT under shaking conditions. Cells were collected with a scraper and washed 3 times with PBS. Cell pellets were resuspended into lysis buffer (1% NP-40, 150 mM NaCl, 10 mM Tris-HCl pH. 7.4, 0.1% protease inhibitors), kept on ice for 20 min and then centrifuged at 14,000 *xg* for 10 min at 4°C. From each lysate, an aliquot was taken and resuspended into 2X Laemmli buffer. The remaining lysates were incubated with High Capacity Streptavidin Agarose Resin (50% slurry, 0.02% sodium azide; Thermo Scientific) on a rotary shaker for 2h at 4°C. Streptavidin resin was washed 3 times in lysis buffer supplemented with 0.1% protease inhibitors, followed by centrifugation at 5,000 *xg* for 5 seconds. After the last washing step, the whole solution was carefully removed and 20 μL of 2X Laemmli buffer was added to the resin to elute the biotinylated cell surface proteins. Samples were either stored at -80°C or used immediately for Western Blot analysis.

### 2.13 Measurement of total H_2_O_2_ production

Hydrogen peroxide (H_2_O_2_) production was measured using AmpliFlu Red reagent (#92001, Sigma-Aldrich). MPM cells (5x10^4^) were seeded onto a 96-well plate and allowed to grow to 90% confluence. To assess total H_2_O_2_ production, the medium was replaced with PBS + 2% BSA + 0.7 mM MgCl_2_ and 0.7 mM CaCl_2_, supplemented with 40 µM Amplex Red reagent, 1 µg/ml horseradish peroxidase, 5 µg/ml superoxide dismutase and 100 µM NaN_3_. Cells were stimulated with HMW-HA (50 µg/ml), LMW-HA (200 µg/ml) and/or C1q (25 µg/ml). After 5 min, fluorescent signal was read at 576 nm with Infinite200 (TECAN).

### 2.14 Proximity ligation assay

Proximity ligation assay (PLA) was performed using the Duolink^®^ *In Situ* Detection Reagents Orange kit (#DUO92007, Sigma-Aldrich). Briefly, MPM primary cells (5x10^4^) were seeded onto round coverslips and allowed to grow to 70% confluence. Cells were fixed with 3% PFA, for 15 min at RT in the dark, and then permeabilized using 0.1% Triton X-100 for 10 min. Non-specific binding was blocked using Duolink Blocking Solution for 1h at RT. Cells were then incubated with primary antibodies diluted in Duolink Antibody Diluent O/N at 4°C. Following washing in Wash Buffer A (10 mM Tris, 150 mM NaCl, 0.05% Tween 20), cells were incubated with secondary antibodies conjugated with PLUS and MINUS probes, for 1h at 37°C. Negative controls included only single primary or secondary antibodies. After two washing steps in Wash Buffer A, cells were incubated with the ligation-ligase solution for 30 min at 37°C, followed by an incubation with amplification-polymerase solution, for over 90 min at 37°C. Cells were washed in the Wash Buffer B (200 mmol/L Tris, 100 mmol/L NaCl) and coverslips were mounted in Fluorescence Mounting Medium with DAPI. Images were acquired using Leica DM3000 microscope (Leica, Wetzlar, Germany) and a Leica DFC320 digital camera.

### 2.15 siRNA-mediated silencing of gene expression

H28 cells (3.5x10^4^ or 1.5x10^5^/well) were cultured in 24-well plate or 6-well plate respectively, for 24h in complete RPMI medium. For transfection, the medium was replaced with PS-free Opti-MEM (Gibco, ThermoFisher) containing 10 nM of predesigned gene-specific siRNAs for scrambled control, *HYAL2*, *CD44* or *C1QBP* (ONTarget SMARTpool Plus, Dharmacon), and lipofectamine RNAiMAX reagent (Invitrogen). Transfection was carried out for 72h in a 5% *v/v* CO_2_ humidified incubator.

### 2.16 Specific blocking of gC1qR

H28 cells (2x10^5^/well) were incubated with 40 µg/mL of F(ab’)_2_ anti-gC1qR blocking antibody diluted in RPMI + 0.5% FBS for 30 min at RT. Cells were then centrifuged, resuspended in RPMI + 10% FBS and seeded onto 24-well plate previously coated with HA or HA+C1q. After O/N incubation, cells were lysed in RNA Lysis Buffer for RNA extraction.

### 2.17 Statistical analysis

Data were analyzed using unpaired one-tailed Student’s t-test. Results were represented as mean ± standard deviation (SD) or standard error mean (SEM). *p <*0.05 were considered statistically significant. All statistical analyses were performed using Prism 9.0 software (GraphPad Software Inc., La Jolla, CA, USA).

## 3 Results

### 3.1 Hyaluronidases are active and variably expressed in malignant pleural mesothelioma

**Figure 1:**
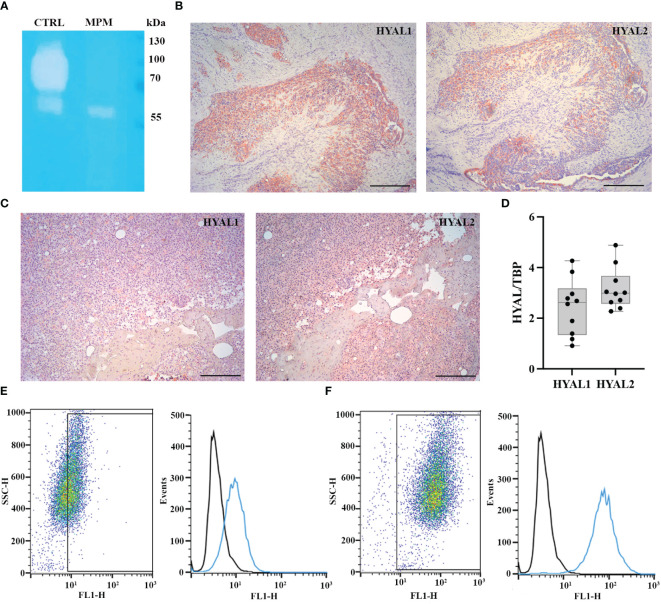
Characterization of hyaluronidase (HYAL) activity, distribution and expression in malignant pleural mesothelioma (MPM). **(A)** Hyaluronic acid zymography confirmed HYALs’ enzymatic activity in MPM. A 10% polyacrylamide gel was impregnated with hyaluronic acid, before running MPM cell lysates under non-reducing conditions. Human serum was used as a positive control for acidic HYAL activity. Gel staining with Alcian blue dye demonstrated a specific HYAL enzymatic activity as suggested by the degradation band detected around 55-60 kDa. **(B, C)** Immunohistochemistry analyses were performed on epithelioid MPM tissue sections of different patients. A positive cytoplasmic and membrane staining for HYAL1 and HYAL2 was detected within the tumour region in all the samples analyzed, despite slightly variable expression levels among different patients (B, higher HYAL1/lower HYAL2; C, lower HYAL1/higher HYAL2). Staining was detected *via* chromogenic AEC (3-amino-9-ethylcarbazole) substrate. Nuclei were stained with Mayer Hematoxylin. Magnification, 100x; scale bar, 100 µm. **(D)** Basal mRNA expression levels of HYALs were evaluated by RT-qPCR in MPM primary cell lysates. Both *HYAL1* and *HYAL2* resulted as highly and variably expressed. TATA-box binding protein (*TBP*) was used as a housekeeping gene to normalize gene expression results. Data were expressed as a mean of experiments performed on ten different MPM populations in triplicates ± standard deviation (SD). **(E, F)** Basal protein expression levels of HYALs were evaluated by flow cytometry. MPM cells were stained for HYAL1 **(E)** and HYAL2 **(F)** and the fluorescence intensity of the cells incubated with primary antibodies (cyan line) was compared with unrelated staining (black line). Pseudocolor plots and histograms report the expression of HYALs in one representative experiment.

We initially aimed at determining the total HYAL enzymatic activity in MPM cells. We carried out a zymographic assay by impregnating a polyacrylamide gel with HA. After running MPM cell lysates under non-reducing conditions, the gel was dipped in an acidic buffer to allow enzyme renaturation and restore activity. Gel staining with Alcian blue highlighted a specific HYAL enzymatic activity as suggested by the degradation band detected around 55-60 kDa (**Figure 1A**). Unfortunately, due to their close MW and similar pH of activity as well as the low sensitivity/specificity of the assay, it was not possible to determine the specific contribution of HYAL1 and HYAL2 to the total enzymatic activity. However, the zymography clearly demonstrated that HYALs were active in MPM cells.

Thus, we examined HYAL expression and distribution in MPM TME *via* immunohistochemical analysis of epithelioid MPM tissue sections, which revealed a cytoplasmic and membrane staining within the tumor region, despite detecting slightly variable expression levels of HYAL1 and HYAL2 among different patients (**Figures 1B, C**). As a general trend, HYAL1 appeared to be more intensely expressed at the tissue level.

To evaluate the basal expression levels of HYAL1 and HYAL2 in MPM primary cells, we performed RT-qPCR using total mRNA extracted from freshly isolated MPM cell populations. Both *HYAL1* and *HYAL2* were found to be highly expressed in the MPM samples (**Figure 1D**), revealing an overall higher expression of HYAL2. We also examined HYAL1 and HYAL2 protein expression levels by performing cytofluorimetric analyses on MPM primary cells. Both HYAL1(**Figure 1E**) and HYAL2 (**Figure 1F**) were found to be expressed in MPM cells.

### 3.2 HYAL2 is widely distributed within MPM cells and is present on the cell membrane

**Figure 2:**
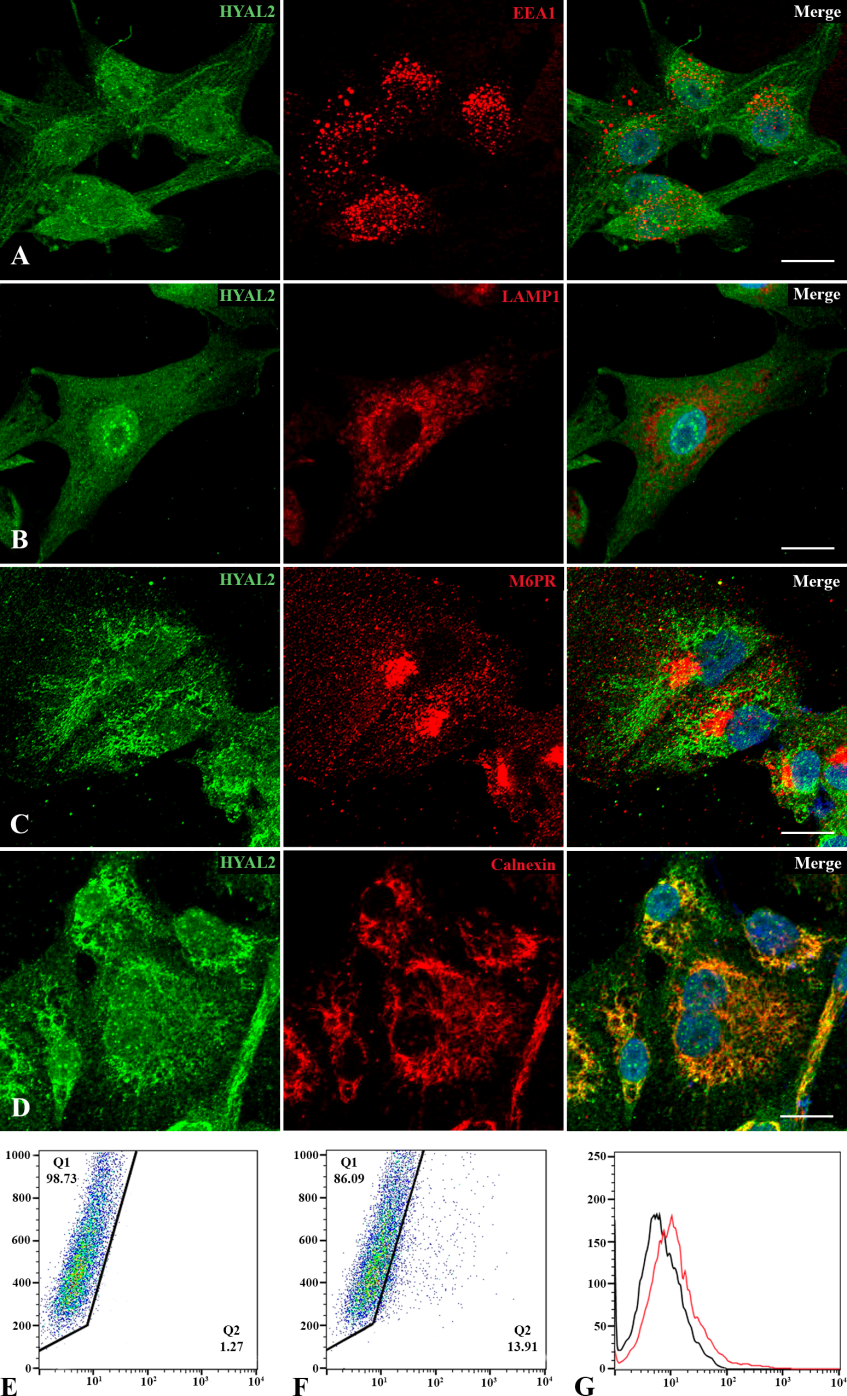
Intracellular and cell surface localization of HYAL2. MPM primary cells were seeded onto rounded coverslips, fixed, permeabilized and stained with human anti-HYAL2 in green and human anti-EEA1 **(A)**, anti-LAMP1 **(B)**, anti-M6PR **(C)** and anti-calnexin **(D)** in red. On the right, the merged fluorescence is shown. Nuclei were stained in blue with DAPI. Scale bar, 10 µm. **(E, F)** The presence of HYAL2 on the cell surface was confirmed by flow cytometry on non-permeabilized cells: secondary antibody alone as control staining **(E)**, compared to HYAL2-stained cells **(F)**. The fluorescence intensity of the cells incubated with HYAL2 (red line) was compared with unrelated staining (black line) **(G)**. Pseudocolor plots and histograms present the expression of HYAL2 in one representative experiment.

Since data on HYAL2 cellular localization are quite conflicting (30), we revisited its intracellular distribution within MPM primary cells. Immunofluorescence microscopy was performed *via* co-immunolabelling of HYAL2 and some organelle-specific markers. Due to the acidic pH required for its activity, we first investigated the co-localization of HYAL2 with specific markers relevant for the endocytic pathway, such as early endosome antigen 1 (EEA1, **Figure 2A**) and lysosomal-associated membrane protein 1 (LAMP1, **Figure 2B**). Surprisingly, confocal microscopy failed to reveal co-localization of HYAL2 and the proteins associated with endocytic pathway. Thus, we turned our attention to the secretory pathway and endoplasmic reticulum (ER)/Golgi markers, testing in particular the co-localization with mannose 6-phosphate receptor (M6PR, **Figure 2C**) and calnexin (**Figure 2D**). Co-labelling with HYAL2 showed a very low signal overlap with the trans-Golgi network marker, M6PR, but an intense co-localization with the ER resident protein, calnexin, as expected for a GPI-anchored molecule such as HYAL2. In order to establish if HYAL2 is localized also on the cell surface of MPM cells, we performed a flow cytometric analysis on live cells (**Figures 2E–G**). Our results confirmed the presence of a plasma membrane HYAL2 fraction, indicating that MPM cells express this enzyme also on their surface.

### 3.3 HYAL2 is a potential prognostic marker in malignant pleural mesothelioma

**Figure 3:**
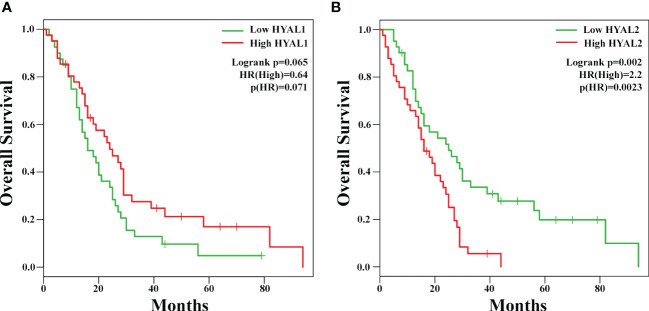
Potential prognostic role of HYAL1 and HYAL2 in malignant pleural mesothelioma (MPM). *HYAL1* **(A)** and *HYAL2* **(B)** mRNA expression levels were correlated to patients’ overall survival by GEPIA2 bioinformatics tool. Kaplan-Meyer plotter were generated using a cohort of 82 MPM patients’ data collected in The Cancer Genome Atlas Mesothelioma (TCGA-MESO) dataset. The cut-off was set at the median of gene expression. *HYAL1* expression did not correlate with MPM patients’ survival rate, whilst high *HYAL2* levels correlated with a lower survival rate of MPM patients (hazard ratio, HR = 2.2; *p* = 0.0023).

To investigate the potential prognostic value of HYALs in MPM, we performed bioinformatics analysis based on gene expression through the survival analysis database GEPIA2, based on a cohort of 82 MPM patients selected by The Cancer Genome Atlas Mesothelioma (TCGA-MESO) dataset. The cohort was divided into two groups according to the median of gene expression. The generation of Kaplan-Mayer plotters did not highlight a significant correlation between HYAL1 mRNA expression and patients’ survival (**Figure 3A**), whereas HYAL2 mRNA expression levels negatively correlated with life expectancy in MPM patients (**Figure 3B**). Thus, the overall survival of patients with high HYAL2 expression appeared to be significantly reduced as compared to patients with its low expression levels (*p* < 0.01).

### 3.4 HA-C1q matrix upregulates the expression of HYAL2

**Figure 4:**
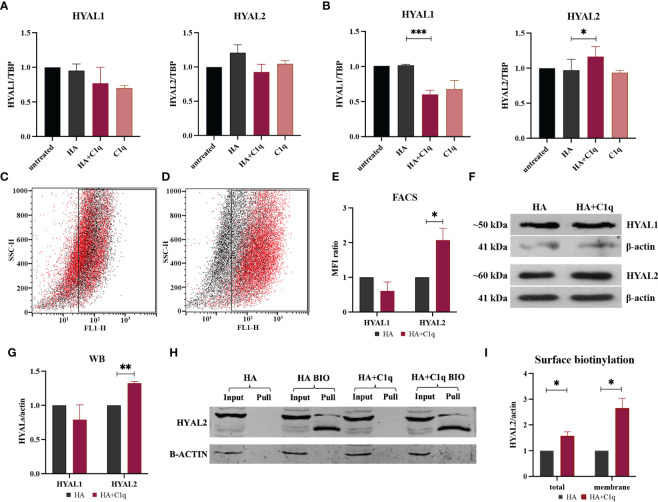
HYAL modulation by HA and/or C1q matrix in malignant pleural mesothelioma (MPM) primary cells. MPM cells were initially treated O/N with HA and/or C1q and total mRNA was analyzed by RT-qPCR **(A)**. MPM primary cells were seeded onto a matrix of HA and/or C1q and HYAL expression was evaluated by RT-qPCR, highlighting a statistically significant *HYAL1* downregulation and *HYAL2* upregulation after HA+C1q treatment as compared to HA alone **(B)**. TATA-box binding protein (*TBP*) was used as a housekeeping gene. Data were expressed as the mean of experiments performed on at least five different MPM populations in triplicates ± SEM. *p < 0.05, ***p < 0.001. **(C-E)** Cytofluorimetric analysis of HYAL1 **(C)** and HYAL2 **(D)** after seeding MPM cells onto HA (black) or HA+C1q (red). **(E)** Histograms represent HYAL1 and HYAL2 expression by cytofluorimetric analysis. Results are expressed considering HA mean of fluorescence intensity as 1 and represent the mean of experiments performed on at least three different MPM populations. The upregulation of HYAL2 was confirmed. *p < 0.05. **(F, G)** Western blot analysis for HYAL expression in MPM cell lysates. Cells were seeded O/N onto HA and HA+C1q matrix, then cell lysates were collected and separated by SDS-PAGE. After transfer, membrane was probed with anti-HYAL1 and anti-HYAL2 primary antibodies and a IRDye 800CW secondary antibody. Signal intensity was detected using an Odyssey CLx near-infrared scanner (LI-COR Biosciences, Lincoln, NE, USA). Image acquisition, processing and data analysis were performed with Image Studio 5.2 (LI-COR Biosciences). Histograms represent the means of experiments performed on at least three different MPM populations. Blots of representative experiments for HYAL1 and HYAL2 **(F)**. β-actin was used to normalize the results. **p < 0.01. **(H, I)** Surface biotinylation assay for the detection of HYAL2 fraction present on the cell surface. MPM cells seeded onto HA+C1q or HA alone were treated with Sulfo-NHS-biotin reagent, isolating biotinylated cell surface proteins upon binding to a Streptavidin-coated resin, and separated on a 10% SDS-PAGE. Membrane was probed with anti-HYAL2 antibody by Western blot analysis. MPM cells not labelled with biotin were processed together with biotinylated samples, in order to control unspecific protein binding to the beads. β-actin was included to normalize the amounts of total lysates. **(I)** Histogram for the quantification of the bands obtained by surface biotinylation assay. *p < 0.05.

Having previously demonstrated an abundance of both HA (27) and the complement protein C1q (17) in MPM TME as well as their synergistic effect on HAS modulation (27), we sought to assess their ability to modulate HYAL expression. Treatment of MPM primary cells with soluble HA and/or C1q did not affect HYAL mRNA expression, as shown in **Figure 4A**. We thus immobilized HA with or without C1q as a matrix, to stimulate MPM primary cells. RT-qPCR analysis revealed that C1q–HA matrix induced a downregulation of *HYAL1* mRNA and a considerable upregulation of *HYAL2* (**Figure 4B**).

HYAL modulation by HA+C1q matrix, as compared to HA alone, was also evaluated at protein level *via* flow cytometry (**Figures 4C–E**) and Western blot (**Figures 4F, G**). These assays failed to confirm a significant HYAL1 downregulation, whereas HA-bound C1q upregulated HYAL2 expression at the protein level, consistent with the RT-qPCR data.

To examine the consequences of the functional interaction between C1q and HA, we investigated HA-C1q-mediated signaling and its impact on plasma membrane trafficking of HYAL2. Thus, we performed surface biotinylation assay on MPM cells seeded onto matrixes after O/N incubation. As shown in **Figures 4H, I**, biotinylation assay confirmed the presence of a HYAL2 fraction on the cell membrane and revealed that HA-C1q matrix brought about HYAL2 trafficking to the cell membrane.

Next, we investigated whether the stimulation with HA and/or C1q was able to induce reactive oxygen species (ROS) production by MPM cells. Thus, MPM cells were stimulated with soluble HMW-HA or LMW-HA, with or without C1q, and the amount of H_2_O_2_ released into the medium was measured. Treatment with soluble LMW-HA, resembling the short HA fragments released in the TME, induced ROS production by MPM cells (**Supplementary Figure 1**), whereas the treatment with HMW-HA and C1q alone was ineffective.

### 3.5 HYAL2 shows a striking co-localization with gC1qR in MPM cells

**Figure 5:**
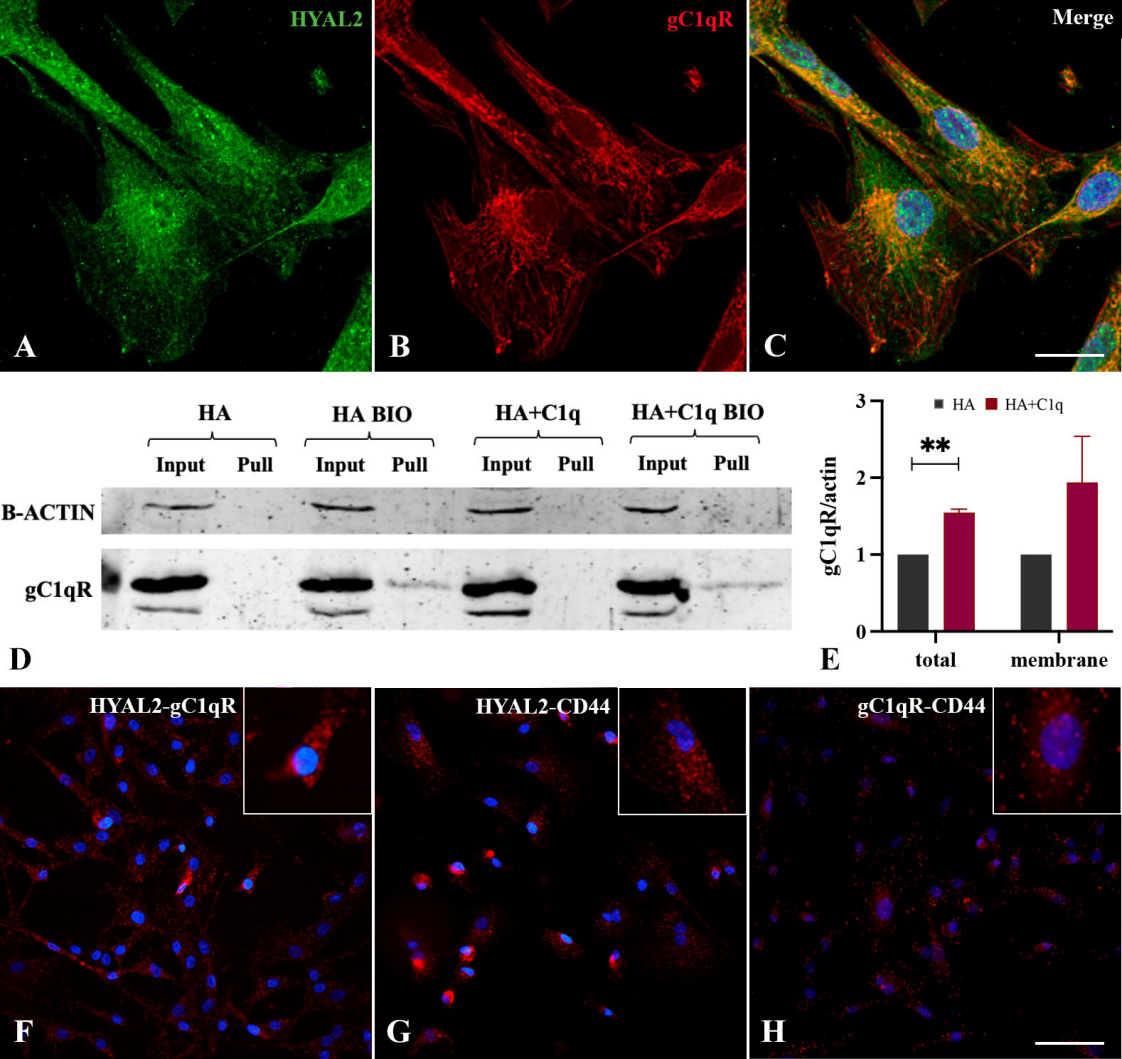
Intracellular and cell surface interaction between HYAL2 and gC1qR. MPM primary cells were seeded onto rounded coverslips, fixed, permeabilized and stained with human anti-HYAL2 **(A)** and human anti-gC1qR **(B)**, determining an intense staining for both proteins. On the right, the merged fluorescence is reported **(C)**. Scale bar, 10 µm. **(D, E)** Surface biotinylation assay for the detection of gC1qR fraction present on the cell surface. MPM cells seeded onto HA+C1q or HA alone were treated with Sulfo-NHS-biotin reagent, isolating biotinylated cell surface proteins upon binding to a Streptavidin-coated resin, and separated on a 10% SDS-PAGE. Membrane was probed with anti-gC1qR antibody by Western blot analysis **(D)**. MPM cells not labelled with biotin were processed together with biotinylated samples, in order to control unspecific protein binding to the resin. β-actin was included to normalize the amount of total lysates. **(E)** Histogram for the quantification of the bands obtained by surface biotinylation assay. **p < 0.01. **(F-H)** Proximity ligation assay for the detection of HYAL2, gC1qR and CD44 interaction. MPM cells seeded onto glass coverslips were fixed, permeabilized and incubated with primary antibodies (anti-HYAL2, anti-CD44, anti-gC1qR), O/N at 4°C. Coverslips were incubated with a pair of oligonucleotide-labeled secondary antibodies (PLA probes), then ligase and DNA polymerase. This allows up to 1000-fold amplified signal that is still tethered to the PLA probe, allowing localization of the signal. HYAL2-gC1qR **(F)**, HYAL2-CD44 **(G)** and gC1qR-CD44 **(H)** interactions were detected. Scale bar, 50 µm.

In view of HYAL2 presence on the cell membrane and its upregulation by HA-C1q in MPM cells, we turned our attention to a potential bridging molecule, gC1qR, which can bind HA as well C1q. Immunofluorescence assay was performed for the simultaneous labelling of HYAL2 and gC1qR in MPM primary cells. Immunofluorescence confocal microscopy revealed a striking co-localization between HYAL2 and gC1qR in MPM cells (**Figures 5A–C**).

To determine a potential interaction between HYAL2 and gC1qR on the cell membrane, we carried out surface biotinylation assay of MPM cells, considering whether HA-C1q matrix could modulate gC1qR expression. The surface labeling of MPM cells with a biotinylated derivative confirmed gC1qR presence on the cell membrane. Similar to HYAL2, MPM cells showed an increased expression of gC1qR, intracellularly and on the cell membrane, when seeded on HA+C1q matrix (**Figures 5D, E**).

To examine a possible physical interaction between HYAL2 and gC1qR, we took advantage of a highly specific and sensitive assay for the detection of endogenous protein-protein interaction, namely Duolink PLA technology. MPM cells seeded onto glass coverslips were fixed, permeabilized and incubated with primary antibodies (anti-HYAL2, anti-CD44, anti-gC1qR). The close proximity between the primary antibodies was demonstrated by the engagement of the conjugated-complementary DNA strands into a rolling circle amplification and the generation of *in situ* fluorescence signal indicating the presence of a protein-protein interaction. The assay allowed the detection of an intense HYAL2-gC1qR interaction visualized by the presence of red dots (**Figure 5F**). Moreover, we confirmed the already known interaction between HYAL2 and CD44 (**Figure 5G**). In addition, we also show here gC1qR-CD44 interaction (**Figure 5H**), suggesting the presence of a tripartite protein complex (HYAL2-CD44-gC1qR).

### 3.6 gC1qR silencing affects HYAL2 expression

**Figure 6:**
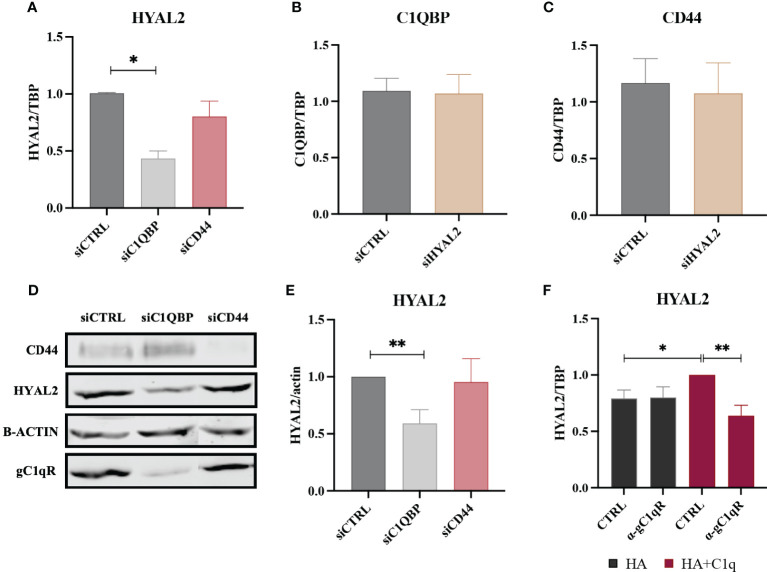
RT-qPCR and Western blot of siRNA-transfected H28 cell line. **(A)** H28 cell line was transfected with siCTRL, siC1QBP or siCD44 for 72h and HYAL2 gene expression was analyzed by RT-qPCR. *HYAL2* mRNA expression was found to be downregulated after *C1QBP* silencing. TATA-box binding protein (*TBP*) was used as a housekeeping gene. Data were expressed as the mean of three experiments performed in duplicates ± SEM. *p<0.05 **(B, C)** H28 cell line was transfected with siCTRL or siHYAL2 for 72h. *C1QBP* and *CD44* mRNA was evaluated, revealing no effects after *HYAL2* silencing. Data were expressed as the mean of three experiments performed in duplicates ± SEM. **(D, E)** Western blot analysis on H28 cell lysates after transfection with siCTRL, siC1QBP and siCD44. Membrane was probed with α-CD44, α-HYAL2 and α-gC1qR primary antibodies and anti-rabbit or anti-mouse IRDye 800CW secondary antibodies. Signal intensity was detected using Odyssey CLx near-infrared scanner. Image acquisition, processing and data analysis were performed by Image Studio 5.2. **(E)** Histograms represent the means of three experiments performed in duplicate. β-actin was used to normalize the results. **p<0.01. **(F)** H28 cells were treated with anti-gC1qR blocking antibodies and seeded onto HA+C1q or HA alone. HYAL2 expression was then evaluated through RT-qPCR. *TBP* was used as a housekeeping gene. *p<0.05; **p<0.01.

To understand interdependence of the tripartite interaction between HYAL2, gC1qR and CD44, we carried out short interfering RNA (siRNA) experiments using H28 (**Figure 6**) and ZL34 cells (**Supplementary Figure 2**), two human MPM cell lines. We initially analyzed *HYAL2* mRNA expression in MPM cell lines silenced for *C1QBP* (gC1qR gene) or *CD44*. Interestingly, we found that the silencing of *C1QBP* caused downregulation in *HYAL2* gene expression as compared to the control, whereas *CD44* silencing did not affect *HYAL2* expression (**Figure 6A**). Interestingly, *HYAL2* silencing had no effect on *C1QBP* (**Figure 6B**) and *CD44* (**Figure 6C**) expression. We were able to validate these observations at the HYAL2 protein level by Western blot (**Figures 6D, E**).

In order to determine whether gC1qR may be functionally involved in the C1q-dependent upregulation of HYAL2, H28 cells were incubated with an anti-gC1qR blocking antibody before seeding them onto HA+C1q or HA alone. As shown in **Figure 6F**, the blocking of gC1qR hampered the upregulation of *HYAL2* mRNA levels when the cells were cultured on HA+C1q, whilst no difference was observed when cells were cultured on HA alone.

## 4 Discussion

An increasingly activated HA metabolism has been frequently associated with cancer progression, requiring both enhanced HA synthesis and degradation (31). In recent years, there has been a growing interest in the cleavage of anti-inflammatory and anti-fibrotic HMW-HA into pro-inflammatory and pro-fibrotic fragments by both HYALs and tissue ROS, which are abundantly present in the TME. In particular, the overexpression of HYALs has been reported during cancer metastasis *in vitro* and *in vivo* (32–34). Here, we demonstrate that HYAL1 and HYAL2 are variably expressed in MPM tissues and primary cells, and they are enzymatically active.

A growing body of research has characterized HYAL2 enzymatic activity and its function-dependent localization. Lepperdinger et al. reported HYAL2 being restricted to only lysosomal compartment due to the acidic pH required for its optimal activity (35). HYAL2 protein has been localized, due to the presence of a GPI anchor, at the cell surface (36) and in lipid rafts (37), but also in cytoplasm (38), mitochondria (39) and nucleus (40). Our co-immunolabelling experiments confirmed the intracellular distribution of HYAL2 in MPM cells, mainly co-localizing with the ER resident protein calnexin, as expected for a GPI-anchored protein. Flow cytometry and surface biotinylation assay also validated HYAL2 presence on the cell membrane.

Interestingly, bioinformatics analysis *via* GEPIA2 tool revealed that high *HYAL2* mRNA expression levels correlated with a poor prognosis in MPM patients. This is consistent with the fact that HYAL2 overexpression and its hyaluronidase activity can generate LMW-HA fragments, which encourage tumor cell proliferation and invasion (41).

HA and the complement protein C1q are abundantly present in the MPM TME (20, 27). We have previously demonstrated that HA-C1q interaction exerts pro-tumorigenic effects (20) and impacts on HA synthesis by regulating the expression of HAS3 (27). In this study, our hypothesis was that the combined effect of HA and C1q could impact on the hyaluronidase activity of MPM tumor cells. Interestingly, HA-bound C1q significantly upregulated the expression of HYAL2 at both mRNA and protein levels. HYAL2 is GPI-anchored, with an intrinsically weak enzymatic activity, which is responsible for the cleavage of HMW-HA polymers into ~20 kDa HA fragments (42). Thus, its increased expression and activity would favor an increase in LMW-HA fragments in the MPM TME, which are potent pro-inflammatory, immunostimulatory and pro-angiogenic molecules. However, HA fragmentation can also be brought about by ROS (43), which are abundantly generated following asbestos fiber uptake during malignant transformation of mesothelial cells, thereby creating a unique inflammatory microenvironment. We showed here that the treatment of MPM cells with LMW-HA, which mimicked the short HA fragments released in the TME, increased ROS production by MPM cells, irrespective of C1q presence. Since Monzon et al. reported that ROS are able to directly stimulate expression of HYAL2 *via* p38MAPK-dependent signaling pathway (44), we can hypothesize a feed-forward loop indirectly triggered by the abundance of C1q in the TME. C1q can increase HYAL2 expression and activity, leading to enhanced production of LMW-HA fragments in the TME; additionally, ROS generation precedes upregulation of HYAL2 expression. The complex interplay is also consistent with the evidence that HA-bound C1q enhances p38 phosphorylation and the consequent activation of the MAPK pathway (20).

Our data provide evidence that HA-bound C1q upregulates HYAL2 transport and/or turnover at the plasma membrane of MPM cells. To identify the chaperone molecule, we came across gC1qR, which is a receptor for HA as well as C1q. The receptor was initially characterized for its ability to bind the globular head domain of C1q (45); subsequent studies revealed its identity as hyaluronic acid binding protein 1 (HABP1) and mitochondrial p32 (46). Co-immunolabelling experiments and PLA highlighted a striking co-localization between HYAL2 and gC1qR. It is reasonable to propose that HYAL2 and gC1qR may be juxtaposed to CD44 on the MPM cell surface, whose interaction is fundamental for HYAL2 catalytic activity (47). Co-localization of gC1qR and CD44 in the lipid rafts during lamellipodia formation has already been reported (48). In fact, gC1qR lacks a consensus motif for a transmembrane domain and relies on signaling partners’ transmembrane domains, such as β1 integrin and CD44 (49, 50). Since CD44 and gC1qR are both essential receptors for HA, their proximity may create high affinity anchorage spots for pericellular HA, rendering it available ffor HYAL2-mediated HA fragmentation. This evidence is also supported by PLA results, which confirmed a physical interaction among the three proteins.

RNA interference experiment revealed a potential regulatory function exerted by gC1qR on HYAL2 expression; in fact, silencing of the *C1QBP* gene downregulated HYAL2 expression both at mRNA and protein levels. This observation strengthened our notion that gC1qR may be critically involved in the signaling triggered by HA-C1q interaction. Thus, we interrogated if the C1q-dependent upregulation of HYAL2 may rely on gC1qR involvement by blocking the receptor with a specific antibody. The functional blockage of gC1qR hindered HA-C1q signaling and prevented HYAL2 upregulation. It is possible that gC1qR, *via* its interaction with HYAL2 and CD44, can undergo conformational change, thus exposing its binding sites for the gC1q domain. C1q present in the TME may then interact with gC1qR, HYAL2 and CD44 complex, and promote signaling.

The novel interaction between gC1qR and HYAL2 unveiled another regulatory role of gC1qR: by acting on HA metabolism, gC1qR may control the key functions of HA in tumor progression and its involvement in most of the hallmarks of cancer, such as sustenance of proliferative signaling and tumor-promoting inflammation, evasion of apoptosis, induction of angiogenesis, promotion of invasion and metastases, deregulation of energy metabolism and evasion of the immune response (4). Our findings lend credence to recent literature which describe gC1qR as an immunoregulator of TME, determining progression and metastatic properties of cancer cells, thus, becoming a novel promising target for immunotherapy (51). Peerschke et al. already demonstrated that the blockage of gC1qR with the monoclonal antibody 60.11 caused a reduction in MPM tumor size due to apoptosis enhancement and decreased neovascularization (26). Thus, therapeutic targeting of gC1qR may be an attractive proposition due to its pivotal role in regulating different aspects of TME, comprising HA metabolism.

## 5 Conclusion

HA-bound C1q enhances HYAL2 expression in MPM cells, suggesting a resultant increased rate of HA catabolism and the release of pro-inflammatory and pro-tumorigenic LMW-HA fragments, which in turn may stimulate MPM cells to release ROS in the TME. These data are consistent with an overall tumor-promoting activity of C1q, in concert with HA, in the MPM TME. The co-localization and physical interaction between HYAL2 and gC1qR (a receptor of both HA and gC1q domain), seem to suggest a potential involvement of gC1qR in HA-C1q signaling, most likely requiring also the contribution of the main HA receptor CD44.

## Data availability statement

The original contributions presented in the study are included in the article/**Supplementary Material**. Further inquiries can be directed to the corresponding authors.

## Ethics statement

The studies involving human participants were reviewed and approved by 34/2016. The patients/participants provided their written informed consent to participate in this study.

## Author contributions

Conceptualization, RB, PZ, CA and AB; methodology, AB and RV; resources, MG, CB, FS, PC, AR, and FZ; data curation, AB, RV, AM, and CA; writing—original draft preparation, AB and AM; writing—review and editing, AB, PZ, BG, UK, and RB; supervision, MC and RB; funding acquisition, MC and RB. All authors contributed to the article and approved the submitted version.
