# Supplementary material for: Complement protein C1q stimulates hyaluronic acid degradation via gC1qR/HABP1/p32 in malignant pleural mesothelioma
Source: Front Immunol. 2023 Jun 2;14:1151194. doi: 10.3389/fimmu.2023.1151194 (PMC10275365; doi:10.3389/fimmu.2023.1151194)
Supplement: Supplementary file 1 [file DataSheet_1.docx]

**Supplementary Material to**

**Complement protein C1q stimulates hyaluronic acid degradation *via* gC1qR/HABP1/p32 in malignant pleural mesothelioma**

Andrea Balduit^1,$,*^, Romana Vidergar^2,$^, Paola Zacchi^2^, Alessandro Mangogna^1^, Chiara Agostinis^1^, Micaela Grandolfo^3^, Cristina Bottin^4^, Francesco Salton^4^, Paola Confalonieri^4^, Andrea Rocca^4^, Fabrizio Zanconati^4,5^, Marco Confalonieri^4^, Uday Kishore^6^, [Berhane Ghebrehiwet](https://pubmed.ncbi.nlm.nih.gov/?sort=date&term=Ghebrehiwet+B&cauthor_id=35991308)^7^, Roberta Bulla^2,*^

^1^ Institute for Maternal and Child Health, Istituto di Ricovero e Cura a Carattere Scientifico (IRCCS), Burlo Garofolo, Trieste, Italy

^2^ Department of Life Sciences, University of Trieste, Trieste, Italy

^3^ Neuroscience Area, International School for Advanced Studies (SISSA), Trieste, Italy

^4^ Department of Medical, Surgical and Health Science, University of Trieste, Trieste, Italy

^5^ Struttura Complessa di Anatomia ed Istologia Patologica, Azienda Sanitaria Universitaria Giuliano Isontina (ASUGI), Trieste, Italy

^6^ Department of Veterinary Medicine, United Arab Emirates University, Al Ain, United Arab Emirates

^7^ Department of Medicine, Stony Brook University, Stony Brook, NY, United States

^$^ These authors have contributed equally to this work and share first authorship.

*** Correspondence:**Andrea Balduit (abalduit@units.it).

Roberta Bulla (rbulla@units.it)

Department of Life Sciences, via Valerio 28, University of Trieste, 34127, Trieste, Italy. Phone +39 040 558 8653; Fax +39 040 558 4023

**Running title:** C1q upregulates HYAL2 *via* gC1qR/p32/HABP1.

**Key words:** malignant pleural mesothelioma; hyaluronic acid; hyaluronidase; C1q; HYAL2; gC1qR/HABP1/p32; reactive oxygen species.

**Supplementary Figures**

**Supplementary Figure 1**. Hydrogen peroxide (H_2_O_2_) production was measured by using AmpliFlu Red reagent on MPM cells stimulated with soluble high-molecular weight HA (HMW-HA), soluble low-molecular weight HA (LMW-HA) and/or soluble C1q, as compared to untreated cells. Data are expressed as a ratio between fluorescence units measured by Tecan (E/I 535nm/595nm) in treated and untreated cells (F.U. = 1). The means of three to five experiments performed in triplicate are reported ± SEM. LMW-HA increased ROS production, regardless of C1q presence. ***p*<0.01; ****p*<0.001.


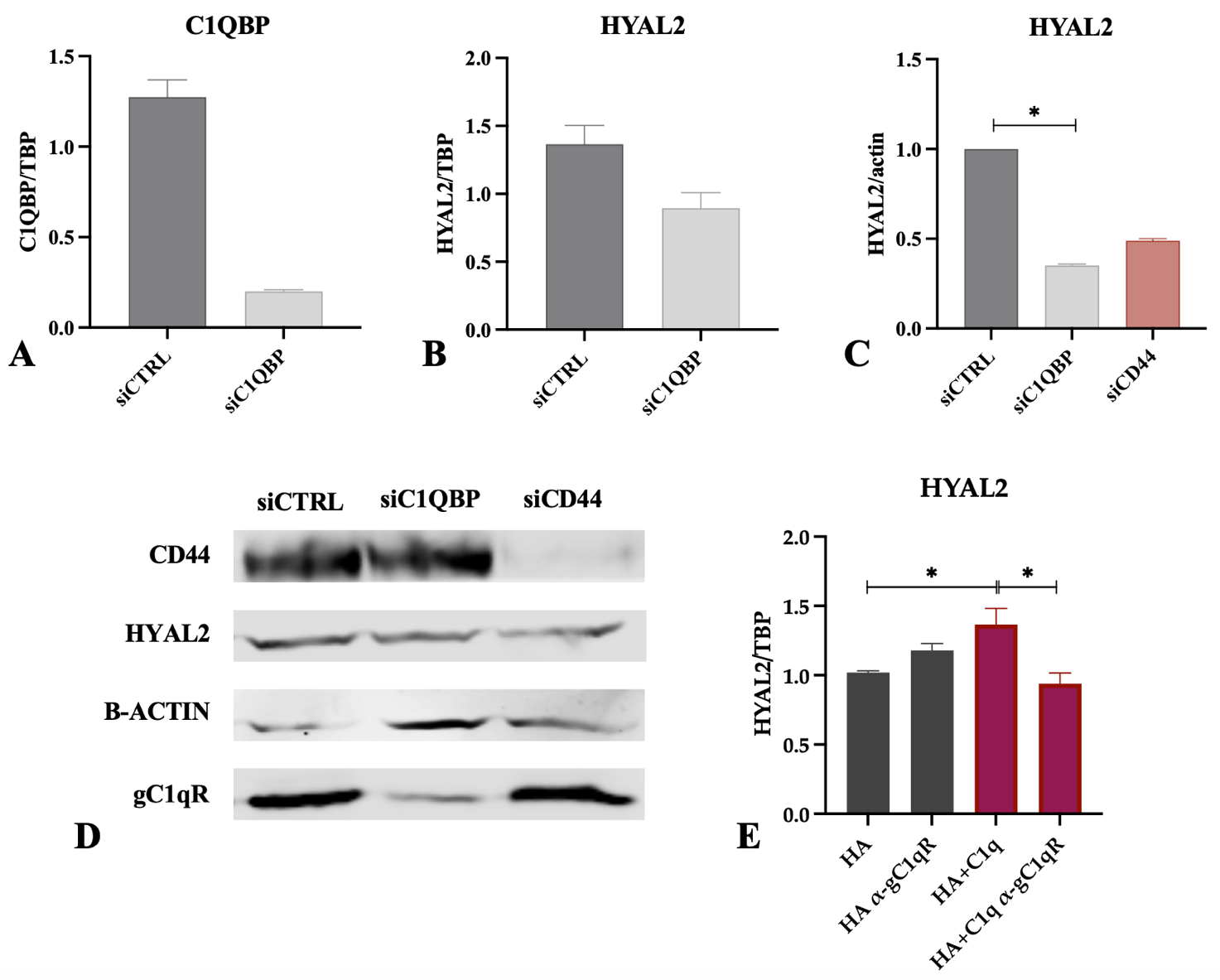


**Supplementary Figure 2**. (**A,B**) ZL34 cell line was transfected with siCTRL and siC1QBP for 72h. *C1QBP* (**A**) and *HYAL2* (**B**) gene expression was analyzed by RT-qPCR. *HYAL2* mRNA expression was found to be downregulated after *C1QBP* silencing. TATA-box binding protein (*TBP*) was used as a housekeeping gene. Data were expressed as the mean of three experiments performed in duplicates ± SEM. (**C,D**) Western blot analysis performed on ZL34 cell lysates after transfection with siCTRL, siC1QBP and siCD44. Membrane was probed with α-CD44, α-HYAL2 and α-gC1qR primary antibodies, followed by anti-rabbit or anti-mouse IRDye 800CW secondary antibodies. Signal intensity was detected using Odyssey CLx near-infrared scanner (LI-COR Biosciences, Lincoln, NE, USA). Image acquisition, processing and data analysis were performed by Image Studio 5.2 (LI-COR Biosciences). Histograms represent the means of three experiments performed in duplicate. Beta-actin was used to normalize the results. **p*<0.05. (**E**) ZL34 cells were treated with anti-gC1qR blocking antibodies and seeded onto HA+C1q, or HA alone. HYAL2 expression was then evaluated through RT-qPCR. TATA-box binding protein (*TBP*) was used as a housekeeping gene. **p*<0.05.
